# Supplementary material for: Multifunctional Silver(I) Complexes with Metronidazole Drug Reveal Antimicrobial Properties and Antitumor Activity against Human Hepatoma and Colorectal Adenocarcinoma Cells
Source: Cancers (Basel). 2022 Feb 11;14(4):900. doi: 10.3390/cancers14040900 (PMC8869984; doi:10.3390/cancers14040900)
Supplement: Supplementary file 1 [file cancers-14-00900-s001.zip › cancers-1589337-supplementary.pdf]

Supplementary Materials

Article

Multifunctional Silver(I) Complexes With Metronidazole Drug Reveal Antimicrobial Properties and Antitumor Activity Against Human Hepatoma and Colorectal Adenocarcinoma Cells

Dominik Żyro, Lidia Radko, Agnieszka Śliwińska, Lilianna Chęcińska, Joachim Kusz, Izabela Korona-Głowniak, Agata Przekora, Michał Wójcik, Andrzej Posyniak and Justyn Ochocki

contents:

Figure S1. Crystals of **$[Ag_2(MTZ)_4]SO_4{\cdot}5H_2O$** (microscopic image)

Figure S3. Crystals of **$[Ag_2(MTZ)_4]SO_4{\cdot}5H_2O$** (real image enlarged)

Figure S3. ^{1}H NMR (600 MHz, DMSO) of metronidazole complex with silver(I) sulfate without **(a)** and with D_20 **(b)**

Figure S4. ^{13}C NMR (600 MHz, DMSO-d_6) of metronidazole complex with silver(I) sulfate

Figure S5. Paper (**a** - upper part) and tissue-paper (**b** - lower part) with applied solutions left in the **dark** and photographed after the time shown

Figure S6. Paper (**a** - upper part) and tissue-paper (**b** - lower part) with applied solutions left in the **daylight** and photographed after the time shown

Figure S7. Paper (**a** - upper part) and tissue-paper (**b** - lower part) with applied solutions left in the **UV-A rays** and photographed after the time shown

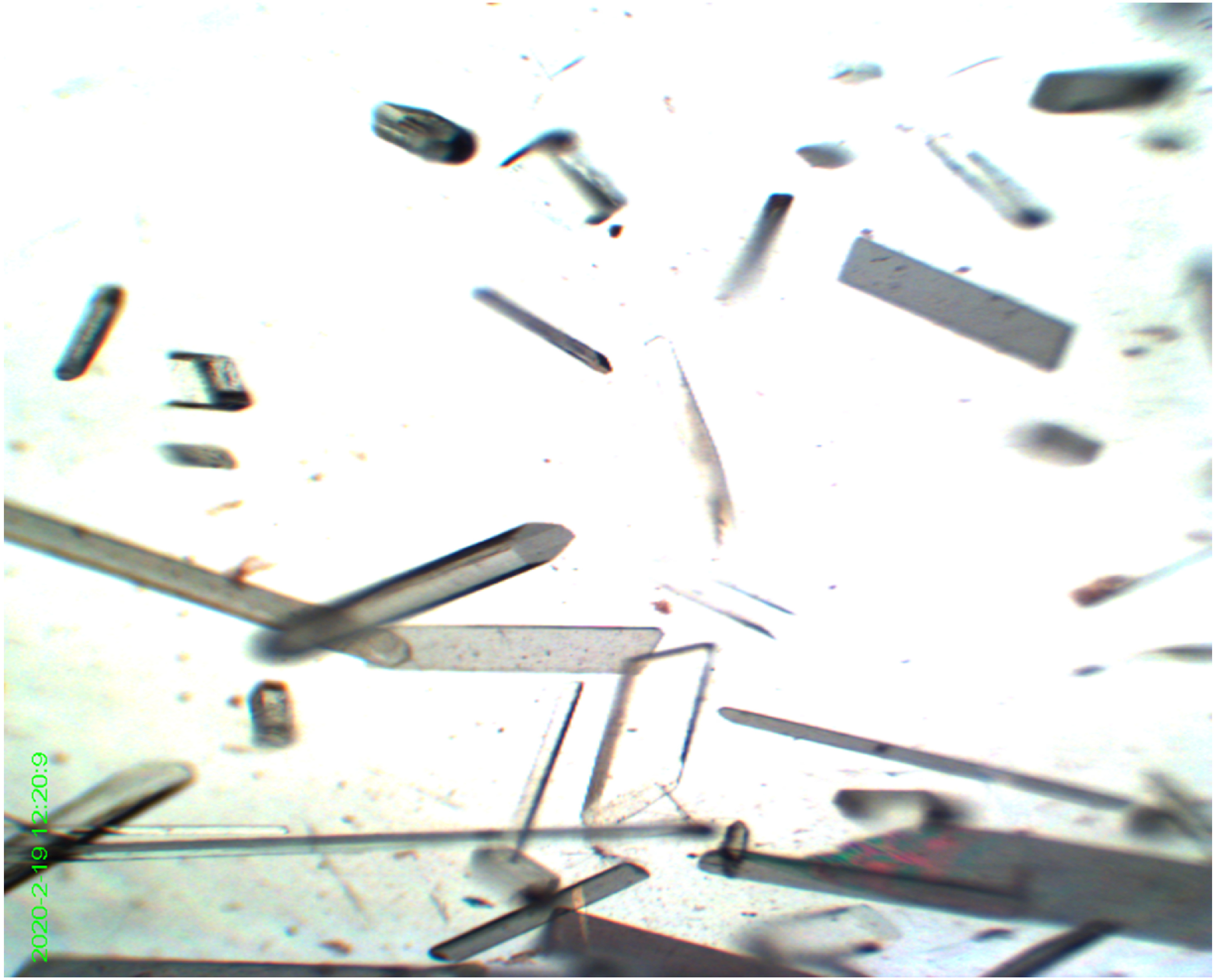

Figure S1. Crystals of **$[Ag_2(MTZ)_4]SO_4{\cdot}5H_2O$** (microscopic image)

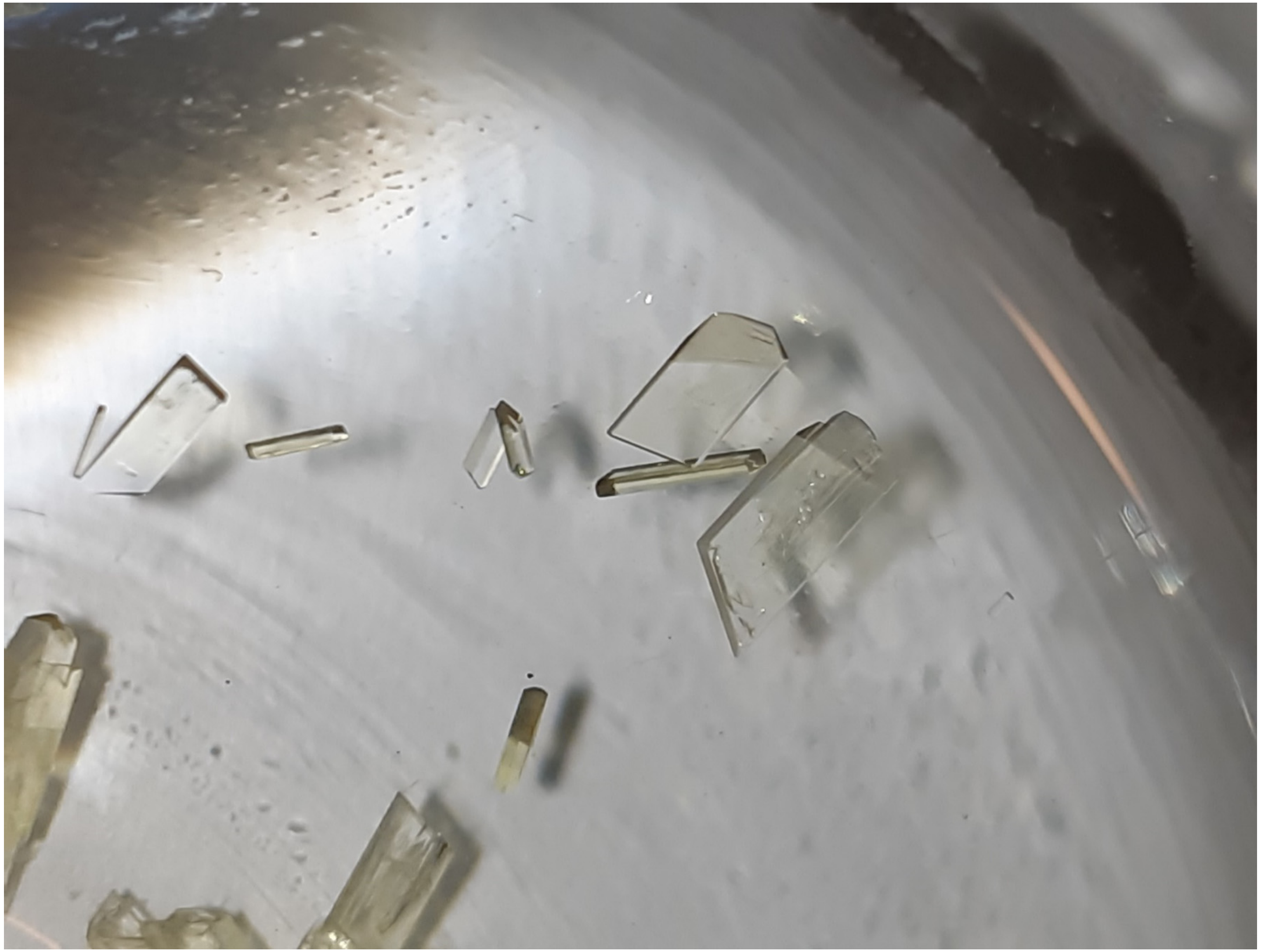

Figure S2. Crystals of **$[Ag_2(MTZ)_4]SO_4{\cdot}5H_2O$** (real image enlarged)

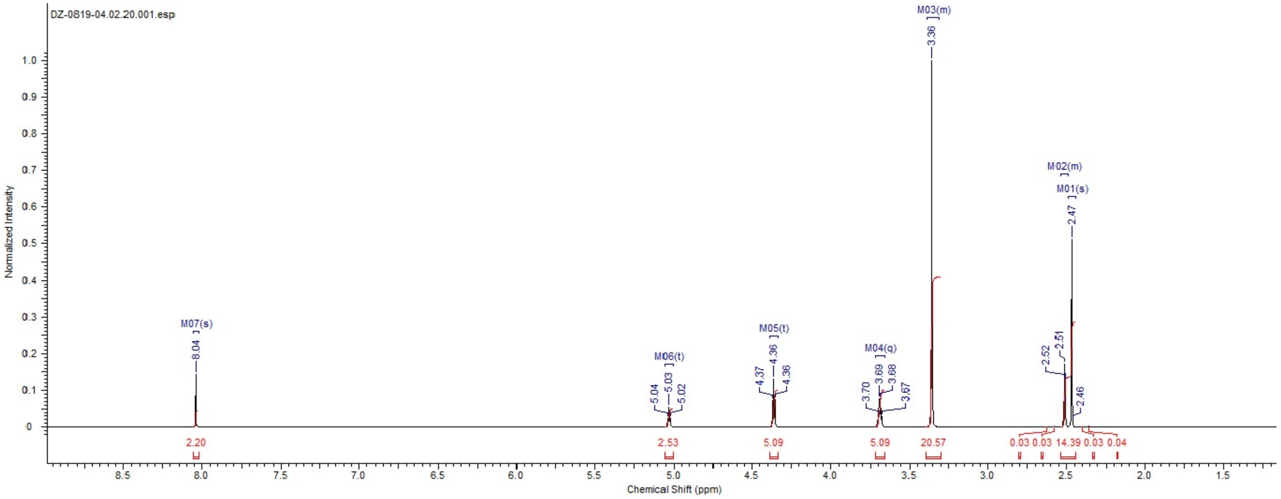

(a)

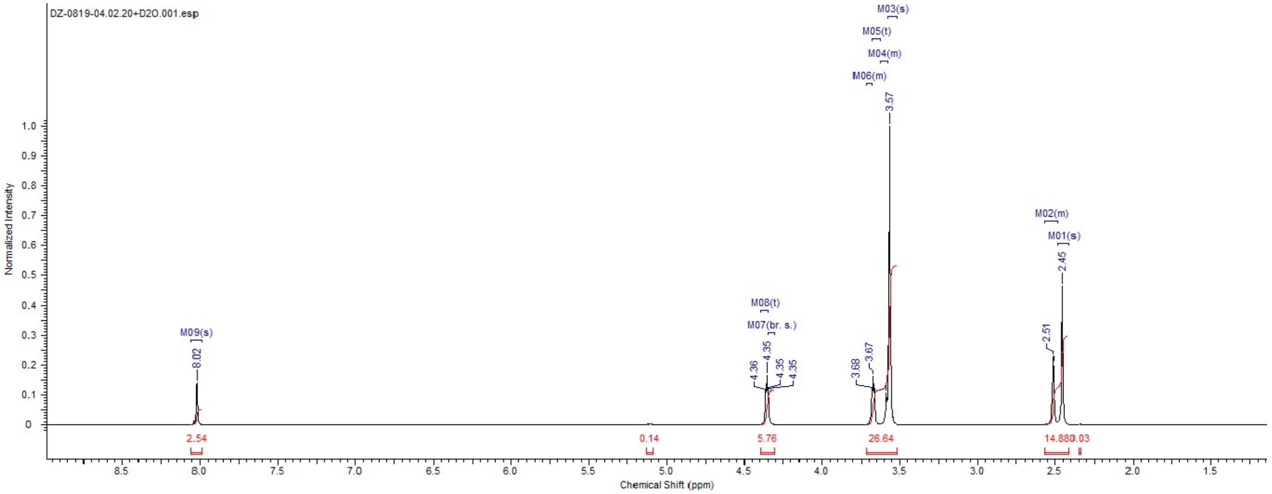

(b)

Figure S3. ^{1}H NMR (600 MHz, DMSO) of metronidazole complex with silver(I) sulfate without (a) and with D_20 (b)

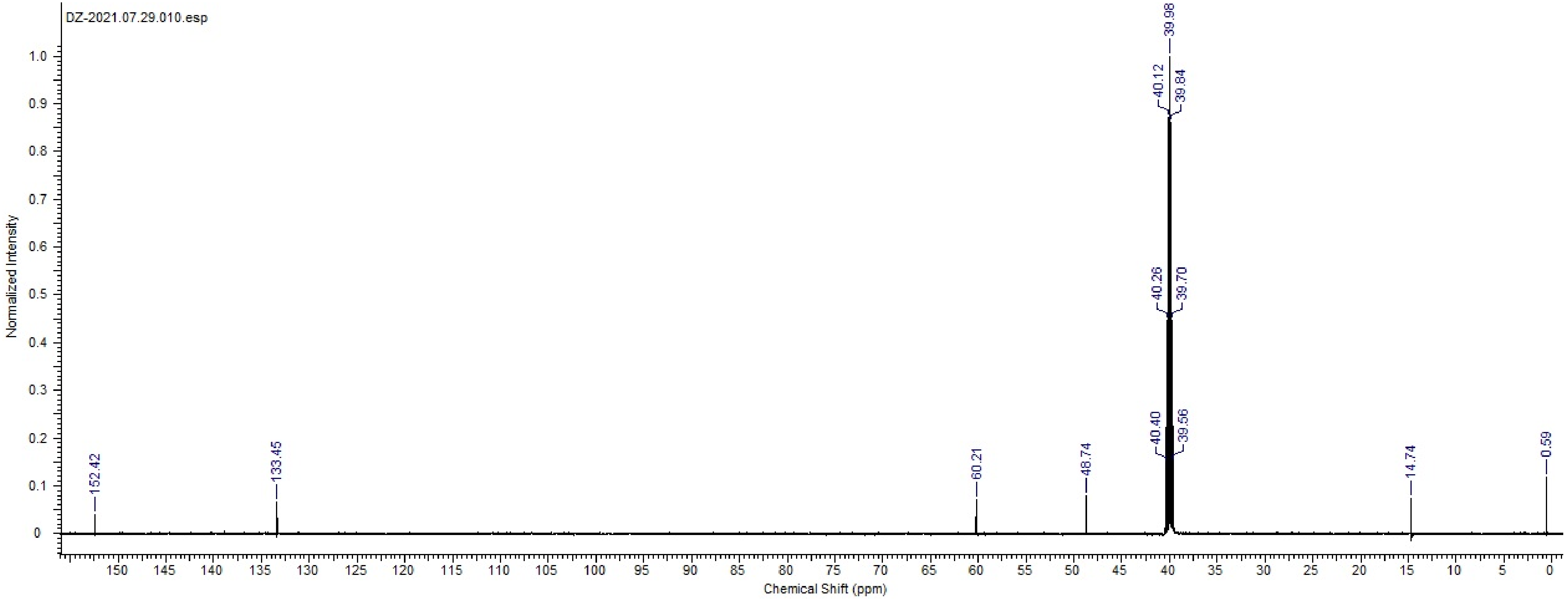

Figure S4. ^{13}C NMR (600 MHz, DMSO-d_6) of metronidazole complex with silver(I) sulfate

(a)

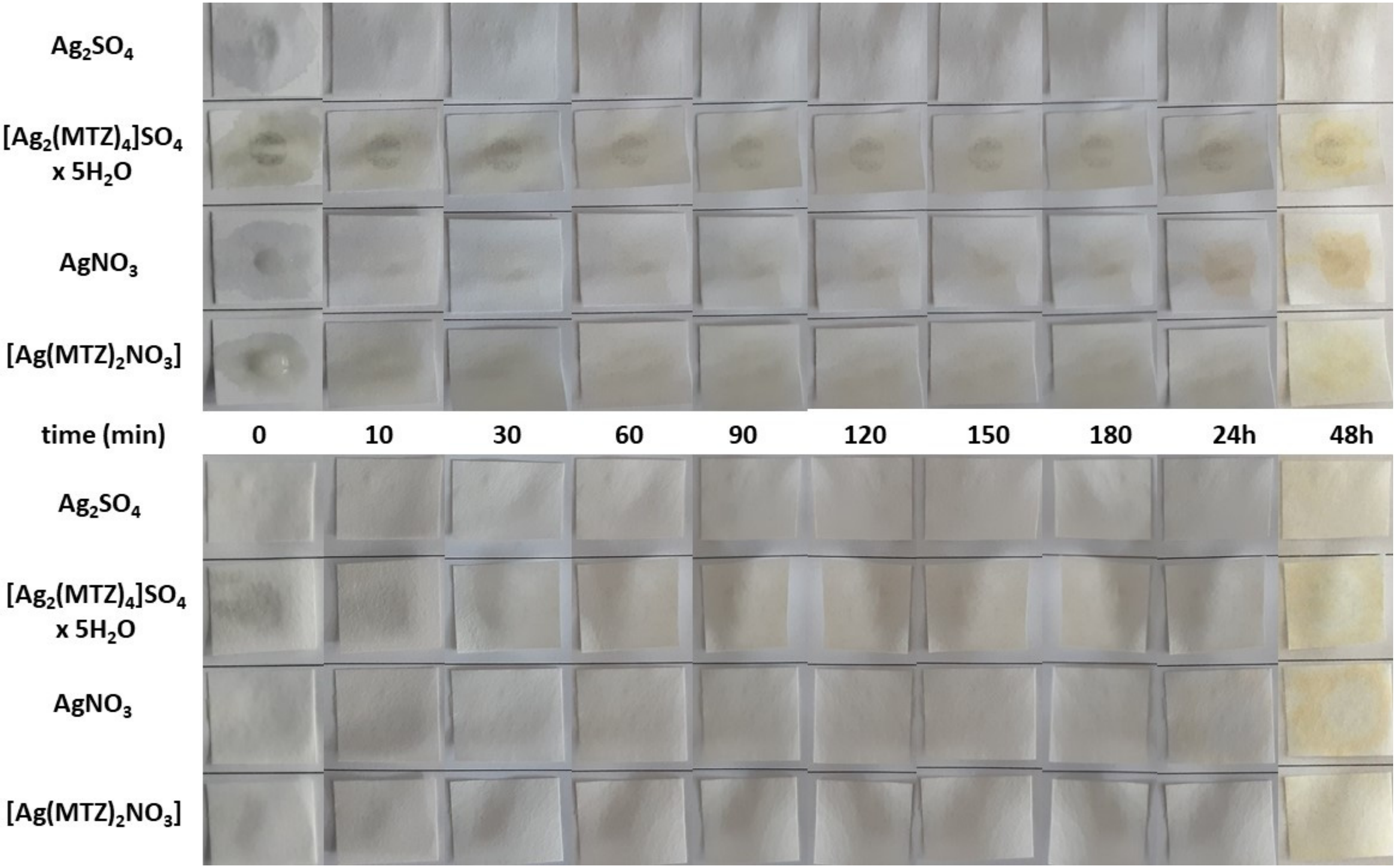

(b)

Figure S5. Paper (**a** - upper part) and tissue-paper (**b** - lower part) with applied solutions left in the dark and photographed after the time shown

(a)

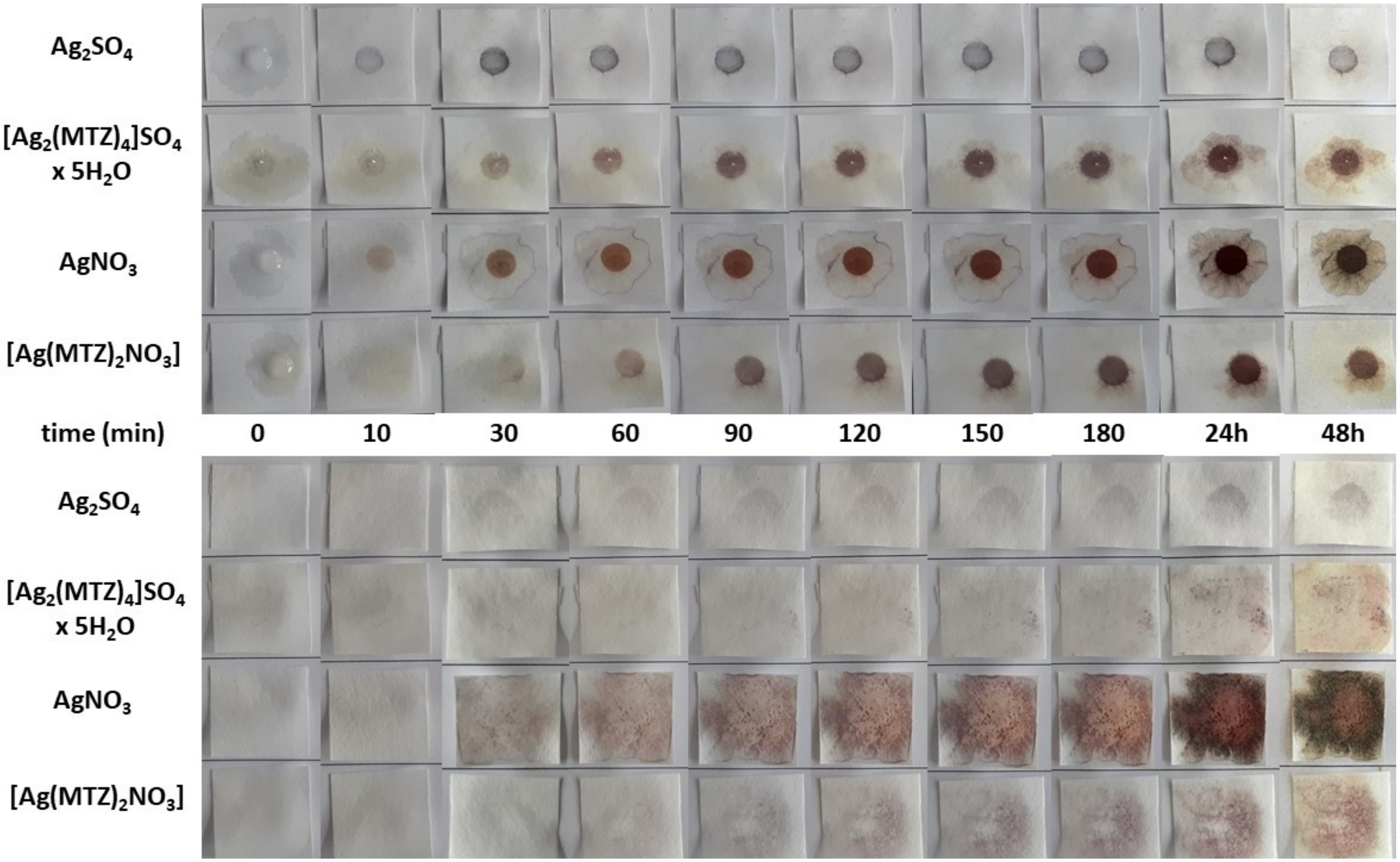

(b)

Figure S6. Paper (**a** - upper part) and tissue-paper (**b** - lower part) with applied solutions left in the **daylight** and photographed after the time shown

(a)

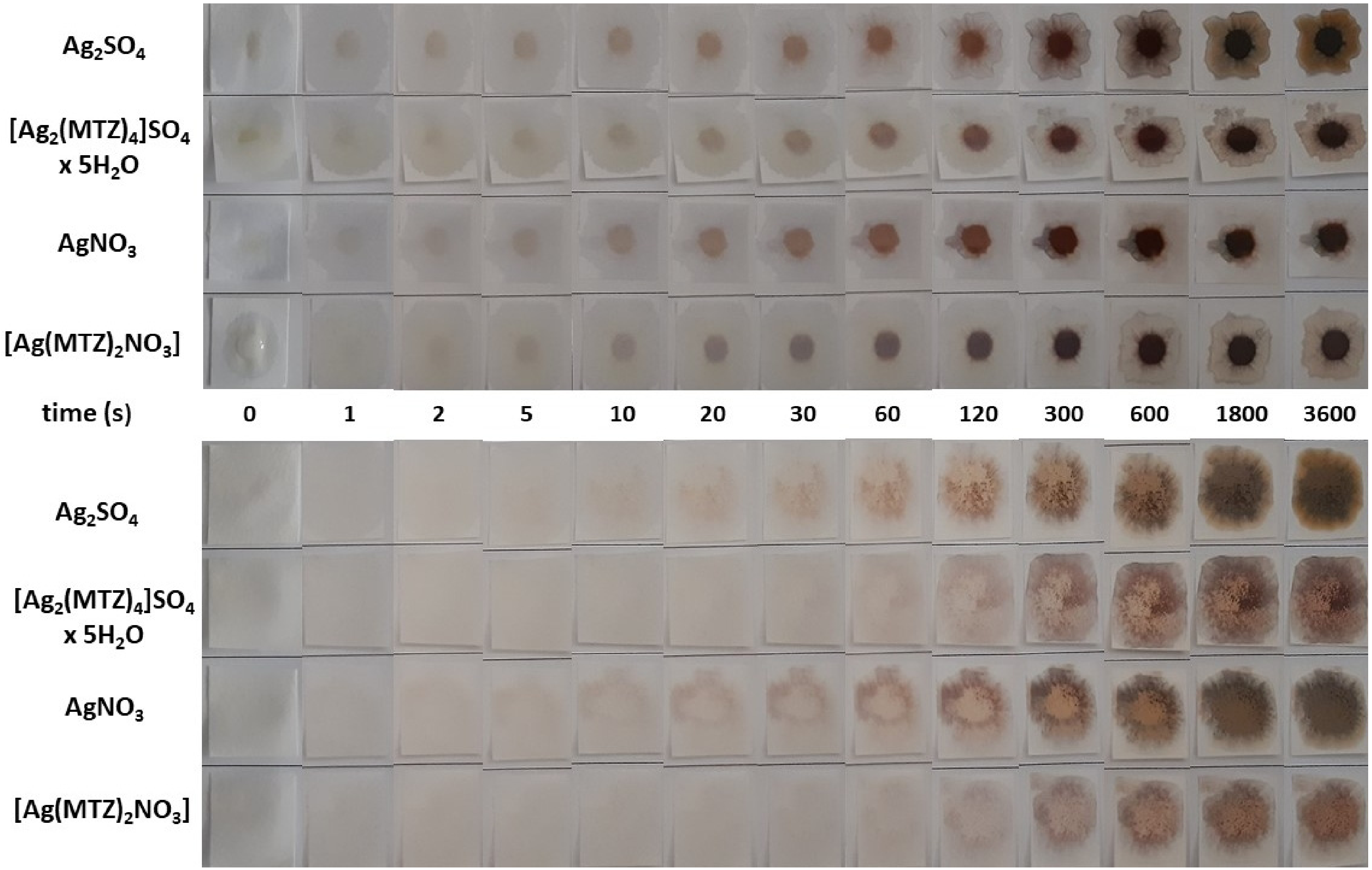

(b)

Figure S7. Paper (**a** - upper part) and tissue-paper (**b** - lower part) with applied solutions left in the **UV-A rays** and photographed after the time shown
